# Variable osteogenic performance of MC3T3-E1 subclones impacts their utility as models of osteoblast biology

**DOI:** 10.1038/s41598-019-44575-8

**Published:** 2019-06-05

**Authors:** Phillip W. Hwang, Jason A. Horton

**Affiliations:** 1 Department of Orthopedic Surgery, SUNY Upstate Medical University, Syracuse, NY 13210 USA; 2 College of Medicine, MD Program, SUNY Upstate Medical University, Syracuse, NY 13210 USA

**Keywords:** Differentiation, Growth factor signalling, Bone development

## Abstract

The spontaneously immortalized murine calvarial cell line MC3T3-E1 and its derivative subclones are widely used models of osteoblast biology. Many investigators have reported conflicting data under seemingly similar experimental conditions, though the specific subclone studied is often not specified. The purpose of this study was to directly compare the commercially available MC3T3-E1 subclones 4, 14, and 24 in terms of responsiveness to osteogenic induction media and/or stimulation with rhPTH[1–34]. We assayed osteogenic gene expression, capacity to deposit and mineralize a collagenous matrix, and the expression and signaling function of PTH1R. Our data demonstrate that each subclone bears little functional resemblance to the others, or to primary calvarial osteoblasts. Specifically, whereas subclone 4 is responsive to PTH stimulation and capable of matrix mineralization, subclones 14 and 24 do not faithfully replicate these key aspects of osteoblast biology. Furthermore, little overlap was observed between the gene expression profile of subclone 4 and primary calvarial osteoblasts. Our experience working with these cell lines demonstrates that the MC3T3-E1 derived cell lines are imperfect models of osteoblast biology, and reinforce the importance of clearly articulating selection and reporting of research materials.

## Introduction

The murine calvarial pre-osteoblast cell line MC3T3-E1 and its derivatives are widely used tools in bone research, having been cited over 4000 times since their introduction in 1981^1,2^. Subsequently, Wang *et al*. isolated a series of 52 single-cell clones derived from the parent MC3T3-E1 cell line^3^. Of these, 10 subclones were selected for further characterization as ‘mineralizing’ (subclones 4, 8, 11, 14, and 26) or ‘non-mineralizing’ (subclones 17, 20, 24, 30, and 35) based on several criteria, including their expression of several hallmark osteoblastic genes (Runx2, Osteocalcin, PTH1R), alkaline phosphatase activity and the ability to deposit mineralized matrix *in vitro* and/or *in vivo*^3^. Several of these subclones are currently available for purchase from various cell repositories, including the American Type Culture Collection (ATCC), who suggest that the ‘non-mineralizing’ clones 24 and 30 are suitable as negative controls for the ‘mineralizing’ clones 4 and 14. Despite the commercial availability of these subclones and knowledge of their resemblance to osteoblastic function, only 1% (Table 1) of published papers using ‘MC3T3-E1’ clearly specified which subclone was studied, or if the cells studied were direct descendants of the heterogeneous line originally isolated by Kodama *et al*.^2^. Studies using MC3T3-E1 cells to examine the role of signaling through the parathyroid hormone receptor (PTH1R) in bone metabolism have been particularly confounding^4–7^. It is possible that this incongruity may be due to heterogeneity of the parent cell line and subsequent phenotypic divergence of its derivative subclones.

**Table 1:** Results from literature search using PubMed, conducted March 5, 2019.

PubMed Search Terms					Search Results (#)		
Base Term	AND				Total	Before 06/01/1999	After 06/01/1999
	E1	Subclone	Clone #	PTH			
MC3T3	–	–	–	–	5035	685	4350
	+	–	–	–	4612	632	3980
	+	+	–	–	43	0	43
	+	+	4	–	31	0	31
	+	+	14	–	15	0	13
	+	+	24	–	5	0	5
	–	–	–	+	180	51	129
	+	–	–	+	170	47	123
	+	+	–	+	2	0	2
	+	+	4	+	1	0	1
	+	+	14	+	0	0	0
	+	+	24	+	0	0	0

Search results are subdivided (from left to right) into: total number of publications; publications before 06/01/199; and publications after 06/01/1999. The 06/01/1999 date corresponds to publication of Wang D, Christensen K, Chawla K, Xiao G, Krebsbach PH & Franceschi RT. Isolation and characterization of MC3T3-E1 preosteoblast subclones with distinct *in vitro* and *in vivo* differentiation/mineralization potential. *J. Bone Miner. Res*. **14**, 893–903 (1999).

The purpose of this study was to compare the response of several commercially available MC3T3-E1 subclones to express osteogenic gene expression, matrix deposition and mineralization in response to induction media and/or PTH[1–34] peptide. We found that subclone 4 demonstrated robust mineralization and responsiveness to rhPTH[1–34], while neither subclone 14 nor subclone 24 displayed these attributes. Furthermore, there was little overlap in the profile of gene expression between the MC3T3-E1 subclones tested and syngeneic primary calvarial osteoblasts maintained in osteogenic differentiation condition. Despite their common origin and commercial availability, our current data demonstrate that variable osteogenic performance of MC3T3-E1 cells is specific to the particular subclone studied. The ability to independently reproduce experimental outcomes from *in vitro* studies is critically dependent upon precise declaration of the cell lines studied, as well as clear identification of all reagents, materials, and media used. Precisely identifying and reporting research materials and reagents are crucial to interpretation and extension of findings between members of the research community.

## Results

### Variable osteogenic response of MC3T3-E1 subclones to differentiation media and rhPTH[1–34]

**Figure 1:**
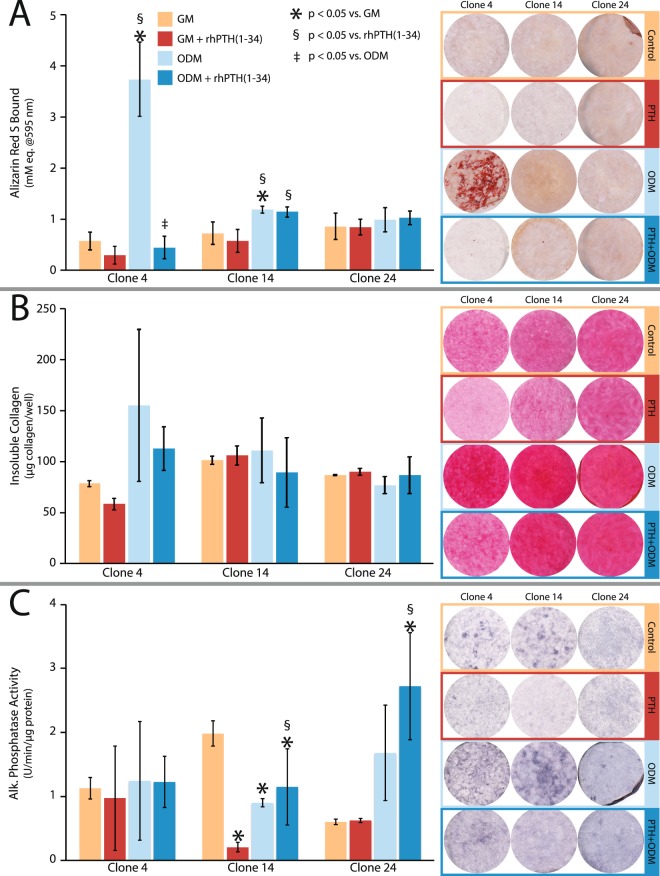
Osteogenic differentiation of MC3T3-E1 subclones 4, 14, and 24 in response to differentiation media and/or intermittent rhPTH[1–34]. Representative histochemical staining (left) and spectrophotometric quantitation (right) of (**A**) matrix calcification with alizarin red S, (**B**) fibrillar collagen deposition with picrosirius red and (**C**) alkaline phosphatase activity. Data shown are mean of n = 3 replicate experiments ± 1 standard deviation. Markers indicate p ≤ 0.05 by ANOVA vs. growth media. Abbreviations: GM = growth media, ODM = Osteogenic differentiation media.

To assess osteogenic function, each of the MC3T3-E1 subclones was cultured in the presence or absence of L-ascorbic acid (100 μM) and β-glycerophosphate (2 mM) for up to 21 days; media was changed at approximately 72 h intervals throughout the experiment. In parallel, the impact of intermittent rhPTH[1–34] treatment on induced osteogenic function was assessed by exposing the cells to 20 nM rhPTH[1–34] for three hours prior to washing and replacing with fresh media. Assays of osteogenic mineral deposition (Fig. 1A) demonstrated that subclones 4 and 14 accumulated significant amounts of calcified matrix in response to differentiation media, whereas subclone 24 did not. Interestingly, the pattern of mineral deposits in subclone 4 occurred primarily in cell-associated nodules, while subclone 14 showed a much more modest and diffuse pattern of staining. Intermittent PTH exposure inhibited mineral deposition in subclone 4, but not subclone 14, suggesting differential responsiveness to this calcitropic peptide hormone. All three subclones deposited an abundance of fibrillar collagen (Fig. 1B) that failed to show any significant response to differentiation media or rhPTH[1–34]. Similarly, each cell line expressed a high basal level of alkaline phosphatase (Fig. 1C). Interestingly, while alkaline phosphatase activity was not affected by either differentiation media or rhPTH[1–34], subclones 14 and 24 showed divergent responses to rhPTH[1–34] alone or in combination with differentiation media.

### MC3T3-E1 subclones express PTH1R, but show variable responsiveness to rhPTH[1–34]

**Figure 2:**
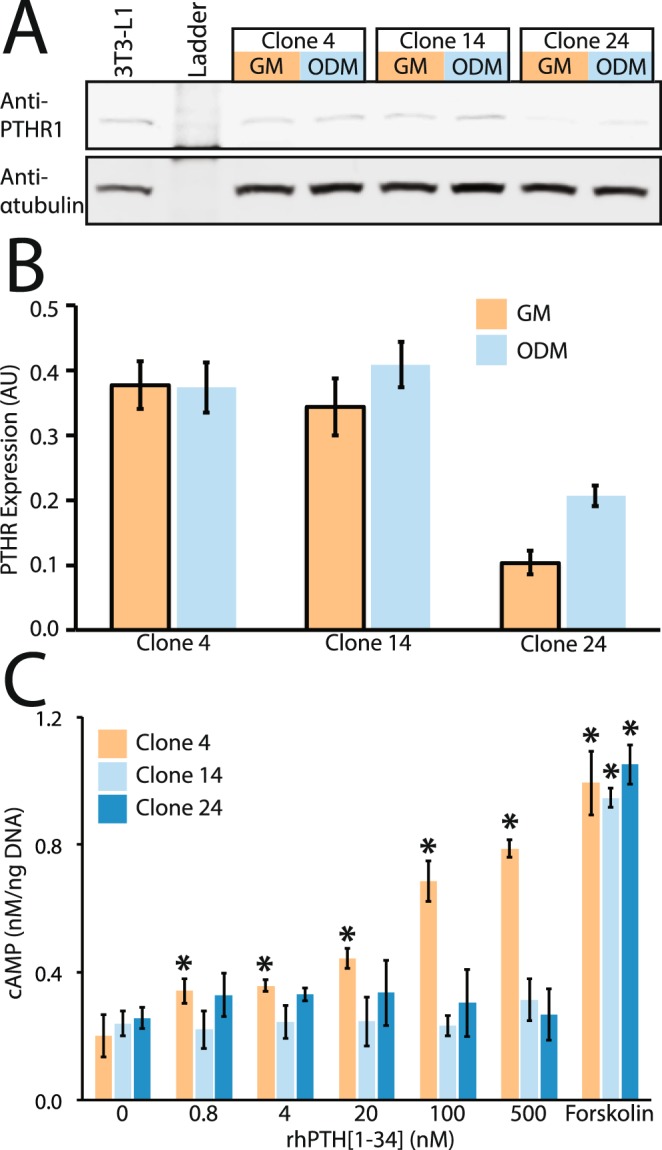
Expression of PTH1R and dose-response relationship of cAMP generation in response to rhPTH[1–34]. (**A**) Representative western blot and (**B**) semi-quantitative densitometry, normalized to α-tubulin showed no significant change in PTH1R expression in response to differentiation media. Image A is a cropped, single channel representation; the two-channel image of the full-length blot is provided as Fig. S1. (**C**) Assay of cAMP generation in response to rhPTH[1–34] treatment, or adenylyl cyclase agonist forskolin. Data shown are mean ± 1standard deviation from n = 3 replicate experiments, normalized to expression of β-actin loading control for western blot or to DNA content for cAMP assay. * indicates p < 0.05 by ANOVA vs. untreated control for each cell line. Abbreviations: GM = growth media, ODM = Osteogenic differentiation media, AU = arbitrary units

Given the variable response to stimulation with rhPTH[1–34] reported in the literature, we considered the possibility that alteration of osteogenic function could be related to differential expression and/or activation of the principal PTH receptor protein (PTH1R) under control or differentiation conditions^8^. Western blotting experiments (Fig. 2A,B) showed that all three subclones expressed PTH1R, whose expression was not affected by exposure to differentiation media for 10 days. We then assayed generation of cAMP, the principal second messenger resulting from ligand-induced binding by G-protein-coupled receptor PTH1R and subsequent activation of adenylyl cyclase (Fig. 2C). Only subclone 4 showed a significant dose-response relationship to treatment with rhPTH[1–34] (p < 0.0001 by linear regression). No relationship between rhPTH[1–34] concentration and cAMP was observed for clone 14 or clone 24 (p > 0.1, linear regression). All three subclones were able to generate cAMP in response to treatment with adenlyl cyclase agonist forskolin (p < 0.0001 vs. vehicle control by ANOVA), indicating that the signaling cascade downstream of PTH1R was intact.

### Distinct profiles of osteogenic gene expression are induced during osteogenic differentiation of and MC3T3-E1 subclones and primary calvarial osteoblasts

**Figure 3:**
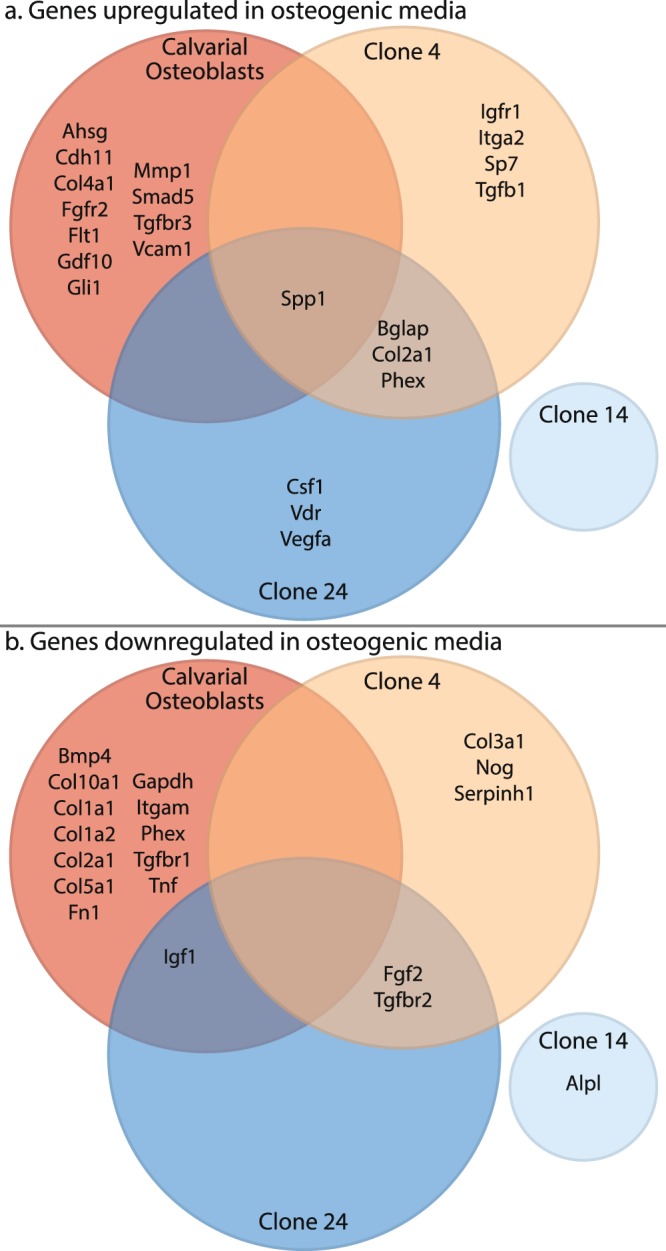
Summary of differential gene expression by MC3T3-E1 sub clones and primary calvarial osteoblasts cultured in differentiation media. (**A**) Up-regulated or (**B**) down-regulated transcripts that were >2-fold and p < 0.05 between control and differentiation media by are shown. Fold-difference data for all 84 genes assayed can be found in Supplementary Table S2. Results of unsupervised hierarchical cluster analysis can be found in Supplementary Fig. S2.

Independent of the PTH/PTH1R mechanism, the divergent osteogenic performance of the MC3T3-E1 subclones in response to differentiation media could be due to intrinsic differences in gene expression or regulation. We evaluated the differential expression of 84 transcripts associated with osteogenesis, in the MC3T3-E1 subclones, as well as in primary murine calvarial osteoblasts (COBs) in response to osteogenic media containing ascorbic acid and β-glycerophosphate to promote matrix deposition and mineralization, respectively. (Figs 3, S2 and Tables 2, S1). Not surprisingly, differentiation media generally induced expression of pro-osteogenic genes in cultures of COBs and MC3T3-E1 subclones 4 and subclone 24 (Fig. 3A). However, there was very little correspondence between COBs and MC3T3-E1 subclones in the specific repertoire of differentially regulated genes in response to differentiation media. There were no genes that shared a common pattern of up- or down-regulation across all MC3T3-E1 subclones and the calvarial osteoblasts. Nonetheless there was some overlap observed between COBs and subclone 4 and/or subclone 24. Osteopontin, a secreted phosphoprotein coded by the *Spp1* transcript, is a key regulator of hydroxyapatite crystal nucleation^9^ that was induced by differentiation media in COBs (4.3-fold), subclones 4 (6.1 fold) and 24 (5.0 fold). Interestingly, osteopontin activity is negatively regulated in the pericellular space by the endopeptidase encoded by the *Phex* gene^10^, which was upregulated in both subclone 4 (6.4-fold) and subclone 24 (11.8-fold), but down-regulated (−4.9 fold) in COB cells. Additionally, differentiation of subclone 4 and subclone 24 were both associated with increased expression of transcripts coding for the extracellular matrix proteins osteocalcin (*Bglap*, 29.4 and 14.4 fold, respectively) and type II collagen (*Col2a1*, 2929.6 fold and 54.1 fold, respectively). Comparatively fewer genes were found to be down-regulated in response to differentiation media, and again showed little correspondence between COBs and the respective MC3T3-E1 subclones. The *IGF1* transcript was down-regulated in both COB (−3.2 fold) and subclone 24 (−4.4 fold).

**Table 2:** Differential gene expression induced by exposure to osteogenic differentiation media for 10 days.

Up-regulated genes			Down-regulated genes		
COB-ODM/COB-GM			COB-ODM/COB-GM		
Gene	Fold Reg.	p-value	Gene	Fold Reg.	p-value
Ahsg	3.6	0.0045	Bmp4	−973.8	<0.0001
Cdh11	3.1	<0.0001	Col10a1	−7.8	<0.0001
Col4a1	2.9	0.0018	Col1a1	−4.6	0.0033
Fgfr2	5.0	0.0013	Col1a2	−2.5	0.0113
Flt1	5.2	0.0004	Col2a1	−5.7	0.0142
Gdf10	10.5	0.0008	Col5a1	−2.5	0.0017
Gli1	3.5	0.0181	Fn1	−2.5	0.0043
Mmp10	4.3	0.0001	Igf1	−3.2	0.0021
Smad5	2.7	0.0426	Itgam	−10.3	0.0013
Spp1	4.3	<0.0001	Phex	−4.9	0.0003
Tgfbr3	8.5	0.0285	Tgfbr1	−2.4	0.0027
Vcam1	2.7	<0.0001	Tnf	−15.0	0.0023
			Gapdh	−2.5	0.0067
**Clone4-ODM/Clone4-GM**			**Clone4-ODM/Clone4-GM**		
Bglap	29.4	0.0476	Col3a1	−2.1	0.0425
Bmp4	2.6	0.0100	Fgf2	−2.6	0.0168
Col2a1	2929.6	0.0366	Nog	−3.3	0.0130
Igf1r	534.9	0.0061	Serpinh1	−2.9	0.0275
Itga2	2.3	0.0366	Tgfbr2	−2.2	0.0130
Phex	6.4	0.0091			
Sp7	2.3	0.0181			
Spp1	6.1	0.0028			
Tgfb1	2.9	0.0199			
**Clone14-ODM/Clone14-GM**			**Clone14-ODM/Clone14-GM**		
—	—	—	Alpl	−6.2	0.0185
**Clone24-ODM/Clone24-GM**			**Clone24-ODM/Clone24-GM**		
Bglap	14.4	0.0016	Fgf2	−2.2	0.0281
Col2a1	54.1	<0.0001	Igf1	−4.1	0.0031
Csf1	2.6	0.0003	Tgfbr2	−2.8	0.0017
Phex	11.8	0.0018			
Spp1	5.0	0.0297			
Vdr	2.7	0.0061			
Vegfa	2.8	0.0318			

Data shown if >2-fold up- or down-regulated and p ≤ 0.05 by Student’s t-test. COB = primary calvarial osteoblast, ODM = osteogenic differentiation media, GM = growth media.

## Discussion

The heterogeneous MC3T3-E1 cell line and several of its single cell-derived subclones have been widely used to study the maturation of pre-osteoblastic cells into a matrix mineralizing osteoblast. Despite the commercial availability of these subclones, few authors adequately state which subclone of this cell line was used in their research, complicating the ability of independent investigators to replicate published studies. Indeed, this study was inspired by a series of experiments performed by our group in which different ‘mineralizing’ subclones of MC3T3-E1 cells yielded wildly different results in essentially identical assay conditions.

We sought to compare the responsiveness of several of the commercially available MC3T3-E1 subclones to a standard differentiation protocol, using ascorbic acid and β-glycerophosphate as supplements to induce osteoblastic differentiation and promote mineralization, respectively^3,11,12^. Ascorbate, a necessary co-factor of prolyl- and lysyl hydroxylase enzymes, is required for procollagen synthesis^13^ and secretion^14^ as well as maturation of the collagen triple helix^15^. Ascorbate has also been suggested to promote MC3T3-E1 proliferation, and expression of PTH1R in osteoblasts^8,16^ in a manner that is temporally associated with collagen deposition. The precise role of β-glycerophosphate is less clear, in part due to the wide range of concentrations or treatment schedules reported in the literature. It is generally assumed that β-glycerophosphate is hydrolyzed by alkaline phosphatase expressed in the peri-cellular space or within extracellular vesicular bodies, leading to cell-associated mineral deposition. Our data do not conform to this assumption though, as we detected high levels of alkaline phosphatase activity in all three subclones, yet only subclone 4 accumulated mineral. Importantly, the differentiation media used in our experiments did not contain dexamethasone, which is often used to promote alkaline phosphatase expression in osteogenic cells of rat or human origin, but may inhibit alkaline phosphatase expression and matrix mineralization by osteoblastic cells of mouse origin^17^. Chung *et al*. provide compelling evidence that that 2 mM β-glycerophosphate is sufficient to support cell-associated mineral deposition, and that higher concentrations result in stochastic precipitation of non-apatitic calcium phosphates^18^. Furthermore, studies by Boskey *et al*. demonstrated that neither alkaline phosphatase nor β-glycerophosphate were necessary to initiate apatitic mineral deposition by cell-secreted microvesicles in cell-free gelatin gels^19,20^, suggesting that other secreted molecules, such as osteopontin, osteocalcin, and PHEX may be involved in regulating apatite crystal nucleation and growth.

Intermittent administration of parathyroid hormone (PTH) or recombinant peptide fragments thereof stimulates bone remodeling, with the greater net effect on bone deposition justifying its use as an anabolic agent^21–23^. The type 1 receptor (PTH1R) for PTH and parathyroid hormone-related peptide (PTHrP) is a G protein-coupled receptor that is highly expressed in bone and kidney, and mediates PTH-dependent regulation of calcium and phosphate homeostasis primarily through activation of adenylyl cyclase^24^. Previous reports have demonstrated regulation by alternative promoters results in expression of several splice variants of the PTH1R gene^25^. Furthermore, post-transcriptional modifications^26–30^ may alter its sub-cellular localization and thus its function in signal transduction in response to endocrine PTH or paracrine PTHrP ligands^31^. In characterizing the subclones derived from the parent MC3T3-E1 line, Wang *et al*.^3^ noted that ascorbate treatment increased expression of several osteoblastic transcripts including *PTH1R*, and that the magnitude of this response generally correlated to mineralization capacity. Curiously, data regarding the expression of *PTH1R* transcript by subclone 14 was not reported. Our data demonstrate that while all three subclones express PTH1R protein, only subclone 4 demonstrated responsiveness to rh[PTH1–34] challenge.

Our data demonstrate that, at the transcriptional level, the commercially available MC3T3-E1 subclones bear little resemblance to primary osteoblastic cells they are intended to model. This may be in part due to heterogeneity between the subclones and gradual divergent evolution from their origin as a primary calvarial osteoblast. In spite of the broad use of the MC3T3-E1 cell line, and its derivative subclones throughout the literature, our data lead us to conclude that these cell lines may be of limited utility for study of bone biology, and that data derived from them should be interpreted cautiously.

## Materials and Methods

All primary data and experimental protocols will be made available upon request from the authors. Detailed information on key reagents, materials, cell lines, instruments and software can be found in supplemental document.

### MC3T3-E1 pre-osteoblastic cell lines and primary calvarial cell culture

Murine calvarial osteoblast cell lines MC3T3E1 subclone 4 (CRL-2593), subclone 14 (CRL-2594) and subclone 24 (CRL-2595) were purchased from ATCC (Manassas, VA) and maintained at 37 °C in a humidified 5% CO_2_ atmosphere, in ascorbate-free αMEM base media supplemented with 10% fetal calf serum, 1% Antibiotic-Antimycotic solution and 1 mM sodium pyruvate. Media was changed at 3–4 day intervals, and cells were passaged with Trypsin/EDTA solution at less than 80% confluence to maintain proliferation. MC3T3-E1 cells were seeded at 2000 cells/cm^2^ for all experiments, and allowed to grow to confluence prior to differentiation experiments described below. Cells were screened for mycoplasma contamination (MycoAlert, Lonza) and used within five passages from the purchased stock.

All experiments involving animal subjects were performed in accordance with relevant guidelines and regulations, under a protocol approved by the Institutional Animal Care and Use committee of Upstate Medical University (PHS Assurance A3514-01). Primary calvarial osteoblasts (COB) were isolated from 6-week old female C57BL/6 J mice (n = 9). Prior to harvest, the mice were maintained in ambient environmental conditions (19–22 °C, 50% relative humidity) with ad libitum access to food and water. Prior to use in experiments, the mice were allowed to acclimate to the local environment for one week after delivery from the vendor. Mice were euthanized by CO_2_ inhalation, and the frontal and parietal bones were aseptically removed and cleaned of soft tissues and trimmed to remove the sutures with a 1 mm margin. Osteoblastic cells were obtained essentially as described by Bakker *et al*.^32^. Briefly, calvarial plates were digested in four 30 minute cycles of collagenase/dispase solution at 37 °C, with extensive washing with PBS between cycles. The digested calvarial fragments were then plated on collagen-coated tissue culture plastic in the media above, further supplemented with 100 μM β-mercaptoethanol and 10 nM dexamethasone for the first 7 days only; thereafter during expansion and in all experiments the primary cells were maintained in ascorbate-free αMEM base media supplemented with 10% fetal calf serum, 1% Antibiotic-Antimycotic solution and 1 mM sodium pyruvate without β-mercaptoethanol or dexamethasone. Cells were allowed to migrate out of the bone chips, with media changed every 3–4 days, until cells reached approximately 80% confluence. At this point, primary cells were harvested with Trypsin/EDTA solution, passed through a 70μm mesh filter to remove bone fragments and plated for experiments at 1 × 10^4^ cells/cm^2^. Each COB sample was generated by pooling the product of three mice at the first passage.

### Demonstration of matrix deposition and mineralization *in vitro*

Matrix deposition and mineralization was induced in confluent cultures of MC3T3-E1 subclones and primary cells by supplementing growth media with 100μM L-Ascorbic acid-2-Phosphate and 2 mM β-glycerophosphate (Sigma) for up to 21 days^18^, without addition of dexamethasone^1^. To study the effect of intermittent PTH1R stimulation on matrix synthesis, mineralization and alkaline phosphatase expression, we added rhPTH[1–34] (20 nM final concentration) or saline vehicle to wells three hours prior to media change on days 0,3,6,9,12,15 and 18. After incubation for 3 hours, the media was aspirated and the wells were washed with PBS to remove residual rhPTH[1–34], and fresh media lacking rhPTH[1–34] was added to each well. For demonstration of calcium mineral deposition, cultures were washed in PBS and fixed with ice-cold 4% phosphate-buffered paraformaldehyde (pH 7.4) for 30 minutes, and stained with 40 mM alizarin red S, pH 4.1 (Sigma) for 20 minutes. After staining the plates were washed extensively in deionized water, and allowed to air dry prior imaging on a flat bed scanner. Mineral-bound dye was eluted with acetic acid as described previously by Gregory^33^, with absorbance measured at 405 nm on a Tecan Infinite M200 microplate spectrophotometer. Absorbance values were interpolated to a standard curve of known alizarin dye concentrations, and expressed as mM equivalent per well.

Deposition of mature fibrillar collagen deposition was determined as originally described by Tullberg-Reinert^34^ with minor modification suggested by Chen^35^. Briefly, cultures were washed with PBS, fixed with Bouin’s solution (Sigma), washed extensively in deionized water, prior to staining with 0.1% sirius red in saturated picric acid (Electron Microscopy Sciences Hatfield, PA) for 1 hour. Subsequently, stained wells were washed with 0.5 M acetic acid to remove non-specifically bound dye, washed again with deionized water and air dried prior to imaging on a flat bed scanner. Subsequently, bound dye was eluted in 0.5 M sodium hydroxide and absorbance measured at 590 nm was interpolated to a standard curve of known concentrations of rat tail collagen (Sigma) to quantify collagen deposition on a per-well basis.

Cellular alkaline phosphatase expression was detected by *in situ* cytochemical staining, or quantified from cell lysates. For *in situ* staining, cultures were washed five times with normal saline (0.9% NaCl), fixed for 5 minutes in 4% paraformaldehyde in normal saline, and washed five times with Tris-buffered saline (TBS, pH 8.0) before staining with a 1:10 dilution of 1-Step NBT/BCIP solution (Thermo Scientific) in TBS for 30 minutes at 37 °C. Subsequently, stained wells were washed extensively in deionized water, air dried and imaged on a flat bed scanner. To quantify cellular alkaline phosphatase activity, cultures were washed several times with normal saline, and lysed in ice cold TBS with 0.5% Triton-X100 for 10 minutes. Subsequently, cell monolayers were scraped, transferred to a microcentrifuge tube, and insoluble was debris pelleted by centrifugation at 21,000× g for 10 minutes at 4 °C. Aliquots of the resulting supernatant were then used to quantify alkaline phosphatase activity using the alkaline phosphatase diethanolamine activity assay kit (Sigma) or total protein concentration by Pierce BCA Protein assay (Thermo Scientific) according the manufacturers’ recommended instructions. Alkaline phosphatase activity was interpolated from absorbance values of a standard curve of known concentrations of calf intestinal alkaline phosphatase and normalized to total protein content, and expressed as Units/μg protein.

### Expression of PTH1R and dose-response to rhPTH[1–34]

Western blotting was performed to assess expression of PTH1R under control and differentiation conditions. Briefly, cells were lysed in RIPA buffer supplemented with HALT protease/phosphatase inhibitor (Thermo Scientific), and lysate supernatants were resolved by SDS-PAGE and transferred to PVDF membranes (Biorad). Following blocking, membranes were probed with antibodies against PTH1R (clone 3d1.1, 1:500, Santa Cruz Biotechnologies) and α-tubulin (Clone YL1/2, 1:2000 Thermo Scientific), and visualized using a Licor Odyssey instrument. Semi-quantitative densitometry was performed using ImageStudio software (Licor Inc.). Similarly prepared lysates of 3T3-L1 (CL-173, ATCC) cells were used as a positive control for blotting experiments. A two-color image of the full-length blot can be found in supplemental Fig. S1.

Dose-responsiveness of MC3T3-E1 cells to stimulation with rhPTH[1–34] was assessed using the cAMP-Glo assay (Promega Corp., Madison WI) according to the manufacturer’s recommended protocol. For these experiments, cells plated at 5000 cells/well in a white-walled, poly-L-Lysine coated 96-well plates (Corning) and incubated overnight in growth media. Dilutions of recombinant human parathyroid hormone fragment 1–34 (rhPTH[1–34]) Sigma were prepared in HBSS buffer with 0.5 mM 3-isobutyl-1-methylxanthine (Sigma) and 0.1 mM Ro-20-1724 (Sigma). Forskolin (1 μM Sigma) was similarly prepared for use as a positive control. Prior to stimulation, cells were three times washed with PBS, and then stimulated with rhPTH[1–34] for one hour at 37 °C; subsequently the cells were lysed and incubated for 10 minutes with cAMP-Glo reagent before recording luminescence using a Tecan Infinite M200 plate reader. To account for well-to-well variability in cell number, 10 μL aliquots of the cell lysates were reserved for assay of DNA content by AccuBlue HS DNA assay (Biotium, Inc.) according to manufacturers recommended protocol.

### Osteogenic gene expression arrays

We studied the effect of differentiation media on expression of 84 osteogenesis-associated genes expression for each MC3T3-E1 clones or primary calvarial osteoblasts, using Mouse Osteogenesis RT^2^ Profiler array panel (#PAMM-026Z Qiagen, Valencia, CA) according to the manufacturer’s recommended protocol. Briefly, confluent cultures were exposed to growth media or differentiation media as described above for 10 days, and total cellular RNA was extracted using RNeasy reagents (Qiagen). Cultures which spontaneously detached from the substrate prior to RNA harvest were excluded from further study. For each replicate sample, 1.5 μg of total RNA per sample was reverse-transcribed to cDNA, and subsequently amplified on a Mastercycler ep Realplex2 instrument (Eppendorf AG, Hamburg, Germany), using a constant threshold across all samples to determine Ct value. Data was normalized to the geometric mean expression of four housekeeping genes (*Actb*, *Gusb*, *B2m* and *HSP90a1*), and relative gene expression (2^ΔΔCT^) was determined and used to calculate fold-change differences between control and differentiated cultures using the Gene Globe Analysis application (http://www.qiagen.com/geneglobe). Statistical significance of differences between control and differentiation conditions for each cell line was accepted when p < 0.05 by Student’s t-test.

## Supplementary information

Supplementary Materials

Table S1
