# Supplementary material for: Variable osteogenic performance of MC3T3-E1 subclones impacts their utility as models of osteoblast biology
Source: Sci Rep. 2019 Jun 5;9:8299. doi: 10.1038/s41598-019-44575-8 (PMC6549152; doi:10.1038/s41598-019-44575-8)
Supplement: Supplementary file 1 — Supplementary Materials [file 41598_2019_44575_MOESM1_ESM.pdf]

Supplementary Materials for:

Variable osteogenic performance of MC3T3-E1 subclones impacts their utility as models of osteoblast biology

Phillip W. Hwang, M.S.

Jason A. Horton Ph.D.*

Department of Orthopedic Surgery
SUNY Upstate Medical University
Syracuse, NY USA 13210

Supplementary Materials

Document S1: Essential materials, reagents, instruments and software.

Figure S1: Full length image of Western blot presented in Figure 2a. Lanes 1: Positive Control - 3T3-L1 lysate, 25µg total cellular protein. Lanes 2 & 9 Chameleon Duo protein standard (Licor); Lanes 3 through 8 were loaded with 25µg total cellular protein from MC3T3-E1 sub clone samples as indicated above each respective lane.

Figure S2: Unsupervised hierarchical clustering of normalized osteogenic gene expression level in primary calvarial osteoblasts (COB) and MC3T3-E1 subclones 4, 14 and 24. Cells were maintained in standard growth media (GM) or osteogenic induction media (DM) for 10 days.

Supplementary dataset (Excel Workbook): Normalized gene expression data, fold regulation and p-values for all 84 genes assayed. Data shown are mean $2^{-\Delta\Delta CT}$ ± 1 standard deviation of n=3 independently replicated experiments. Data were normalized to geometric average expression of housekeeping genes Actb, B2M, Gusb and Hsp90ab1.

Materials and Reagents

MC3T3-E1 pre-osteoblastic cell lines and primary calvarial cell culture

Murine cell lines ATCC (Manassas, VA)

MC3T3-E1 Subclone 4, (CRL-2593); RRID: CVCL_5440

MC3T3-E1 subclone 14, (CRL-2594)); RRID: CVCL_5437

MC3T3-E1 subclone 24, (CRL-2595)); RRID: CVCL_5438

Cell culture media & supplements

Ascorbate-free αMEM base media, #A10490 Life Technologies, Grand Island, NY

Fetal calf serum, Lot#A03G001, Gemini Bio-products, W. Sacramento, CA

Antibiotic-Antimycotic solution, #15240 Life Technologies

Sodium pyruvate #S8636, Sigma, St. Louis, MO

0.25%Trypsin/0.1% EDTA #25-053-CI Corning Life Sciences, Corning, NY

Dulbecco's Phosphate Buffered Saline, # 21-031-CM Corning Life Sciences

β-mercaptoethanol #21985023 Life Technologies

L-Ascorbic acid 2-phosphate sesquimagnesium salt hydrate, #A8960, Sigma

Dexamethasone,water soluble, #D2915, Sigma

Collagenase/Dispase solution (0.1U/0.8U/ml) #11097113001, Roche/Sigma

β-glycerophosphate Sigma #G9422)

recombinant human PTH[1-34] peptide, #P3796, Sigma

MycoAlert Mycoplasma Detection Kit #LT07-418, Lonza Bioscience, Walkersville, MD

Histochemical Reagents

Paraformaldehyde #158127 Sigma.

Alizarin red S #A5533 Sigma

Bouin's solution (Sigma # HT10132)

0.1% sirius red in saturated picric acid, # 2635702 Electron Microscopy Sciences

Hatfield, PA

Acetic Acid, ReagentPlus #A6283 Sigma

Sodium Hydroxide 0.5M sodium hydroxide, # 415413 Sigma

Collagen, Type I solution from rat tail, #C3867

Sodium Chloride #S7653 Sigma

Tris-buffered Saline pH 8.0, #T6664, Sigma

1-Step NBT/BCIP Solution, #34042, ThermoFisher Scientific

Triton X-100, #11332481001, Sigma

Alkaline phosphatase diethanolamine activity assay kit #AP0100, Sigma

Pierce BCA Protein assay #23225, ThermoFisher Scientific

<u>RNA isolation and RT-PCR Array analysis</u>

RNEasy Plus Mini Kit, #74134, Qiagen, Valencia, CA

Qiashredder columns, #79654 Qiagen

RT^2 First Strand Kit #330404, #330404, Qiagen

Mouse Osteogenesis RT^2 Profiler array, #PAMM-026Z Qiagen

<u>Cell lysis and Immunoblotting</u>

RIPA Lysis buffer, #89900 ThermoFisher Scientific

HALT Protease/Phosphatase Inhibitor cocktail, #78440 ThermoFisher Scientific

3T3-L1 cell lysate, #sc-2243, Santa Cruz Biotechnology, Dallas, TX

Pierce BCA Protein Assay # 23225m ThermoFisher Scientific

4xProtein Loading buffer #928-40004, Li-Cor, Inc., Lincoln NE

NuPage Reducing Agent , #NP-0009 ThermoFisher Scientific

Mini-Protean TGX SDS-PAGE Gels, 4-20% Gradient #456-1093, Bio-Rad Laboratories, Hercules, CA

Tris/Glycine/SDS buffer #1610732, BioRad

Transblot Turbo LF-PVDF membranes #107-4275, Bio-Rad

Revert Total Protein Stain, #926-11021, Li-Cor, Inc.

Odyssey Blocking Buffer #927-40000, Li-Cor, Inc.

anti-αTubulin, Rat IgG_{2a}, Clone YL1/2, # MA-1-80017, ThermoFisher Scientific

anti-PTH/PTHrP-R, Mouse IgG_1 Clone 3D1.1, #sc-12722, Santa Cruz Biotechnology

IRDye® 800CW Donkey anti-Mouse IgG, #926-32212, Li-Cor, Inc.

IRDye® 680RD Goat anti-Rat IgG, #926-68076, Li-Cor Inc.

cAMP Assay

cAMP-Glo Assay Kit, #V1501 Promega Corp. Madison WI

3-isobutyl-1-methylxanthine #I5879, Sigma

Ro-20-1724, #557502, Sigma

Forskolin, #F3197, Sigma

AccuBlue HS DNA assay, #31006, Biotium, Inc. Fremont, CA

Key Instruments

Infinite M200 Microplate Spectrophotometer/Luminometer, Tecan Group, Ltd.

Männedorf, Switzerland

Mastercycler ep Realplex2 instrument, Eppendorf AG, Hamburg, Germany

Odyssey CLx Imaging System, Li-Cor Inc.

Key software Applications

Gene Globe Analysis application (http://www.qiagen.com/geneglobe)

ImageStudio Lite v5.2.5, Li-Cor Inc.

Supplemental Figures

Figure S1

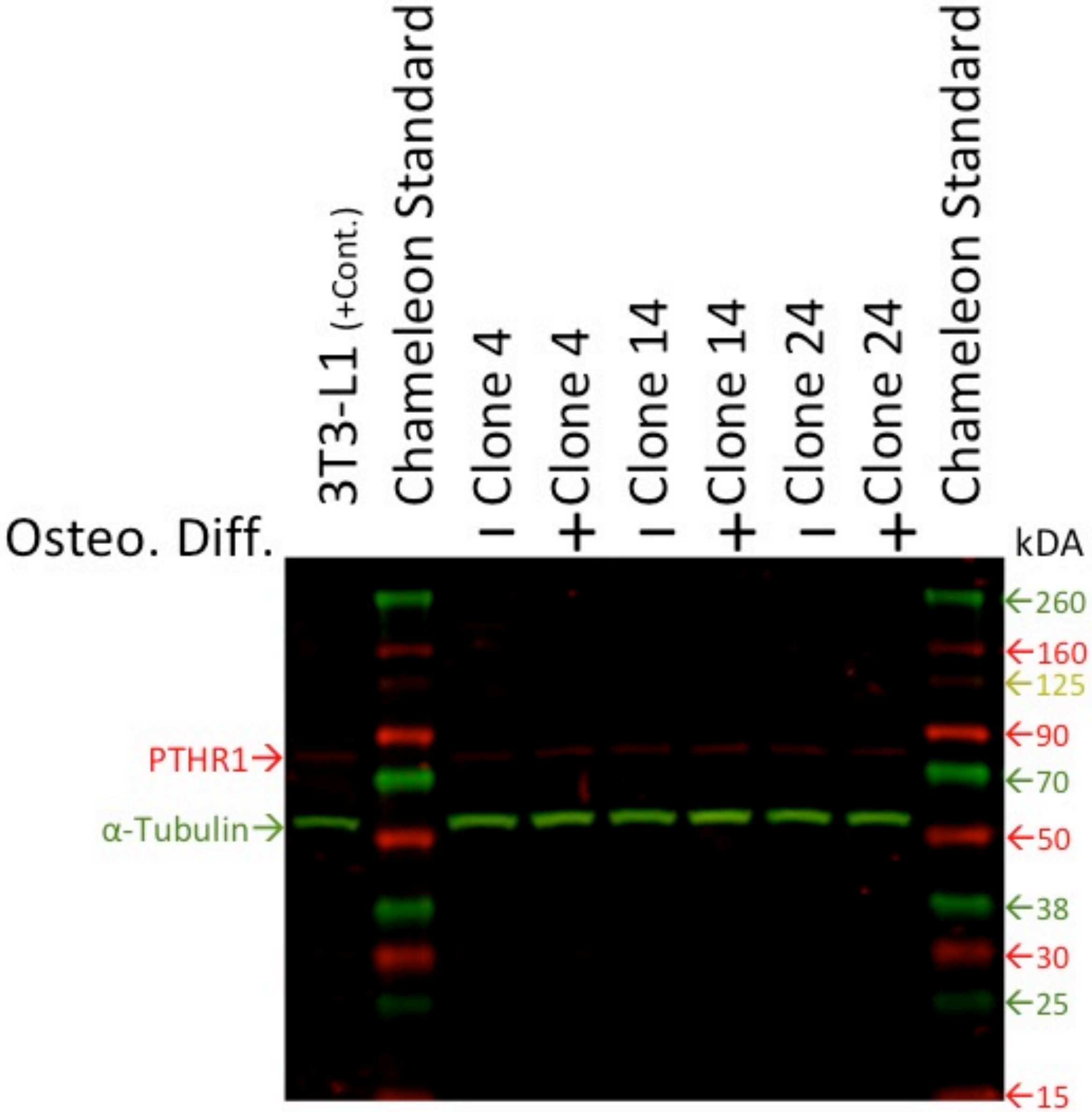

Figure S1: Full length image of Western blot presented in Figure 2a. Lanes 1: Positive Control - 3T3-L1 lysate, 25µg total cellular protein. Lanes 2 & 9 Chameleon Duo protein standard (Licor); Lanes 3 through 8 were loaded with 25µg total cellular protein from MC3T3-E1 sub clone samples as indicated above each respective lane.

Figure S2

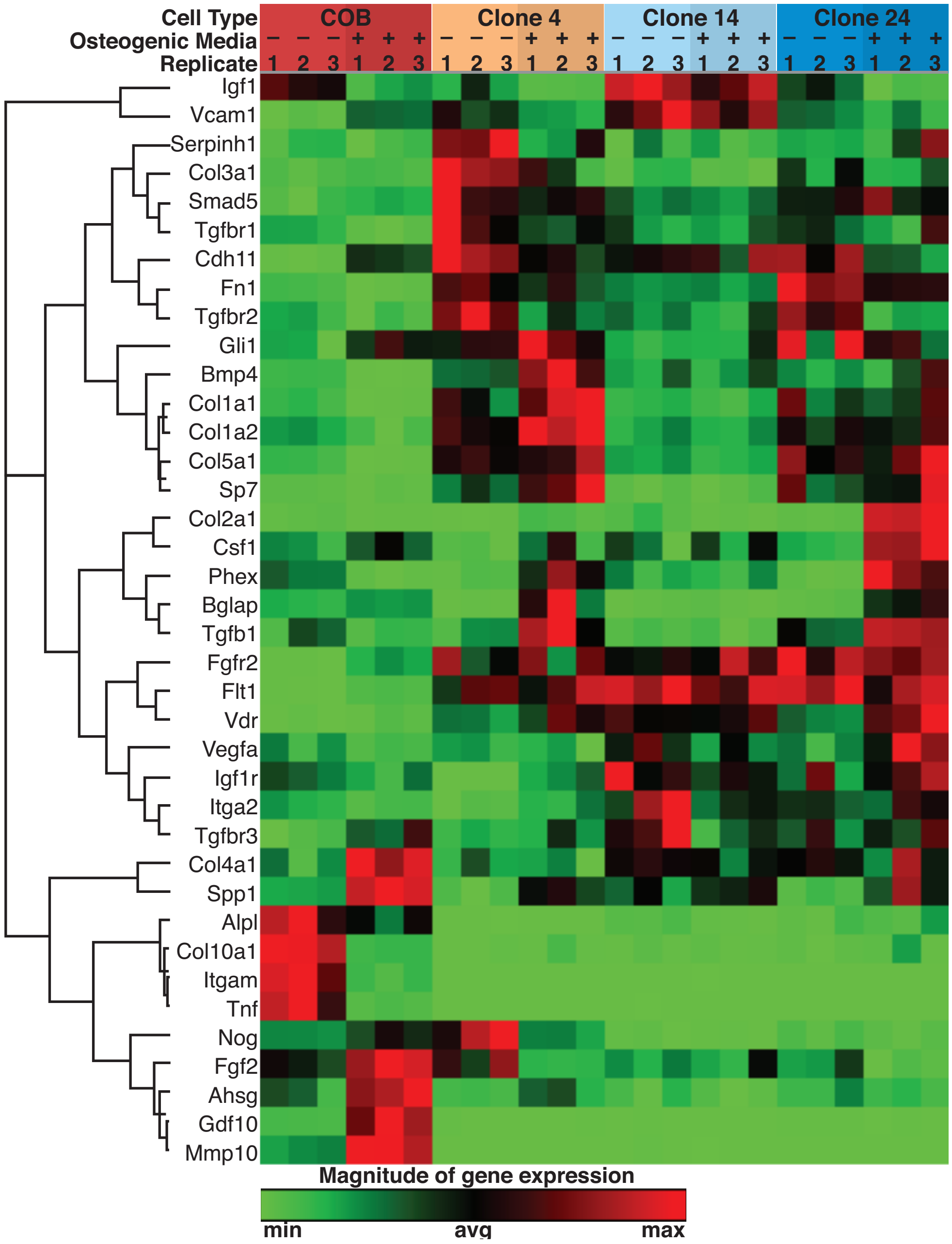

Figure S2: Unsupervised hierarchical clustering of normalized osteogenic gene expression level in primary calvarial osteoblasts (COB) and MC3T3-E1 subclones 4, 14 and 24. Cells were maintained in standard growth media (GM) or osteogenic induction media (DM) for 10 days.

ARRIVE

The ARRIVE Guidelines Checklist

Animal Research: Reporting In Vivo Experiments

Carol Kilkenny[1], William J Browne[2], Innes C Cuthill[3], Michael Emerson[4] and Douglas G Altman[5]

[1]The National Centre for the Replacement, Refinement and Reduction of Animals in Research, London, UK, [2]School of Veterinary Science, University of Bristol, Bristol, UK, [3]School of Biological Sciences, University of Bristol, Bristol, UK, [4]National Heart and Lung Institute, Imperial College London, UK, [5]Centre for Statistics in Medicine, University of Oxford, Oxford, UK.

	ITEM	RECOMMENDATION	Section/ Paragraph
Title	1	Provide as accurate and concise a description of the content of the article as possible.	
Abstract	2	Provide an accurate summary of the background, research objectives, including details of the species or strain of animal used, key methods, principal findings and conclusions of the study.	
**INTRODUCTION**			
Background	3	a. Include sufficient scientific background (including relevant references to previous work) to understand the motivation and context for the study, and explain the experimental approach and rationale.<br>b. Explain how and why the animal species and model being used can address the scientific objectives and, where appropriate, the study's relevance to human biology.	
Objectives	4	Clearly describe the primary and any secondary objectives of the study, or specific hypotheses being tested.	
**METHODS**			
Ethical statement	5	Indicate the nature of the ethical review permissions, relevant licences (e.g. Animal [Scientific Procedures] Act 1986), and national or institutional guidelines for the care and use of animals, that cover the research.	
Study design	6	For each experiment, give brief details of the study design including:<br>a. The number of experimental and control groups.<br>b. Any steps taken to minimise the effects of subjective bias when allocating animals to treatment (e.g. randomisation procedure) and when assessing results (e.g. if done, describe who was blinded and when).<br>c. The experimental unit (e.g. a single animal, group or cage of animals).<br>A time-line diagram or flow chart can be useful to illustrate how complex study designs were carried out.	
Experimental procedures	7	For each experiment and each experimental group, including controls, provide precise details of all procedures carried out. For example:<br>a. How (e.g. drug formulation and dose, site and route of administration, anaesthesia and analgesia used [including monitoring], surgical procedure, method of euthanasia). Provide details of any specialist equipment used, including supplier(s).<br>b. When (e.g. time of day).<br>c. Where (e.g. home cage, laboratory, water maze).<br>d. Why (e.g. rationale for choice of specific anaesthetic, route of administration, drug dose used).	
Experimental animals	8	a. Provide details of the animals used, including species, strain, sex, developmental stage (e.g. mean or median age plus age range) and weight (e.g. mean or median weight plus weight range).<br>b. Provide further relevant information such as the source of animals, international strain nomenclature, genetic modification status (e.g. knock-out or transgenic), genotype, health/immune status, drug or test naïve, previous procedures, etc.	

Housing and husbandry	9	Provide details of:<br>a. Housing (type of facility e.g. specific pathogen free [SPF]; type of cage or housing; bedding material; number of cage companions; tank shape and material etc. for fish).<br>b. Husbandry conditions (e.g. breeding programme, light/dark cycle, temperature, quality of water etc for fish, type of food, access to food and water, environmental enrichment).<br>c. Welfare-related assessments and interventions that were carried out prior to, during, or after the experiment.	
Sample size	10	a. Specify the total number of animals used in each experiment, and the number of animals in each experimental group.<br>b. Explain how the number of animals was arrived at. Provide details of any sample size calculation used.<br>c. Indicate the number of independent replications of each experiment, if relevant.	
Allocating animals to experimental groups	11	a. Give full details of how animals were allocated to experimental groups, including randomisation or matching if done.<br>b. Describe the order in which the animals in the different experimental groups were treated and assessed.	
Experimental outcomes	12	Clearly define the primary and secondary experimental outcomes assessed (e.g. cell death, molecular markers, behavioural changes).	
Statistical methods	13	a. Provide details of the statistical methods used for each analysis.<br>b. Specify the unit of analysis for each dataset (e.g. single animal, group of animals, single neuron).<br>c. Describe any methods used to assess whether the data met the assumptions of the statistical approach.	
RESULTS			
Baseline data	14	For each experimental group, report relevant characteristics and health status of animals (e.g. weight, microbiological status, and drug or test naïve) prior to treatment or testing. (This information can often be tabulated).	
Numbers analysed	15	a. Report the number of animals in each group included in each analysis. Report absolute numbers (e.g. 10/20, not 50%[2]).<br>b. If any animals or data were not included in the analysis, explain why.	
Outcomes and estimation	16	Report the results for each analysis carried out, with a measure of precision (e.g. standard error or confidence interval).	
Adverse events	17	a. Give details of all important adverse events in each experimental group.<br>b. Describe any modifications to the experimental protocols made to reduce adverse events.	
DISCUSSION			
Interpretation/ scientific implications	18	a. Interpret the results, taking into account the study objectives and hypotheses, current theory and other relevant studies in the literature.<br>b. Comment on the study limitations including any potential sources of bias, any limitations of the animal model, and the imprecision associated with the results[2].<br>c. Describe any implications of your experimental methods or findings for the replacement, refinement or reduction (the 3Rs) of the use of animals in research.	
Generalisability/ translation	19	Comment on whether, and how, the findings of this study are likely to translate to other species or systems, including any relevance to human biology.	
Funding	20	List all funding sources (including grant number) and the role of the funder(s) in the study.	

References:
1. Kilkenny C, Browne WJ, Cuthill IC, Emerson M, Altman DG (2010) Improving Bioscience Research Reporting: The ARRIVE Guidelines for Reporting Animal Research. *PLoS Biol* 8(6): e1000412. doi:10.1371/journal.pbio.1000412
2. Schulz KF, Altman DG, Moher D, the CONSORT Group (2010) CONSORT 2010 Statement: updated guidelines for reporting parallel group randomised trials. *BMJ* 340:c332.
